# *Drosophila* Condensin II subunit Chromosome-associated protein D3 regulates cell fate determination through non-cell-autonomous signaling

**DOI:** 10.1242/dev.133686

**Published:** 2016-08-01

**Authors:** Lindsey R. Klebanow, Emanuela C. Peshel, Andrew T. Schuster, Kuntal De, Kavitha Sarvepalli, Madeleine E. Lemieux, Jessica J. Lenoir, Adrian W. Moore, Jocelyn A. McDonald, Michelle S. Longworth

**Affiliations:** 1 Department of Cellular and Molecular Medicine, Lerner Research Institute, Cleveland Clinic Foundation, Cleveland, OH 44195, USA; 2 Bioinfo, Plantagenet, ON K0B 1L0, Canada; 3 Department of Molecular Biosciences, Northwestern University, Evanston, IL 60201, USA; 4 Disease Mechanism Research Core, RIKEN Brain Science Institute, Wako, Saitama 351-0198, Japan; 5 Division of Biology, Kansas State University, Manhattan, KS 66506, USA

**Keywords:** dCAP-D3, KNOT, EGFR, Serum response factor, dSRF, Vein development

## Abstract

The pattern of the *Drosophila melanogaster* adult wing is heavily influenced by the expression of proteins that dictate cell fate decisions between intervein and vein during development. dSRF (Blistered) expression in specific regions of the larval wing disc promotes intervein cell fate, whereas EGFR activity promotes vein cell fate. Here, we report that the chromatin-organizing protein CAP-D3 acts to dampen dSRF levels at the anterior/posterior boundary in the larval wing disc, promoting differentiation of cells into the anterior crossvein. CAP-D3 represses KNOT expression in cells immediately adjacent to the anterior/posterior boundary, thus blocking KNOT-mediated repression of EGFR activity and preventing cell death. Maintenance of EGFR activity in these cells depresses dSRF levels in the neighboring anterior crossvein progenitor cells, allowing them to differentiate into vein cells. These findings uncover a novel transcriptional regulatory network influencing *Drosophila* wing vein development, and are the first to identify a Condensin II subunit as an important regulator of EGFR activity and cell fate determination *in vivo*.

## Abstract (precis)

**Summary:** The chromatin-organizing protein CAP-D3 maintains EGFR signaling in the developing *Drosophila* wing disc, identifying a key transcriptional regulatory network influencing vein development.

## INTRODUCTION

Cell fate decisions are often regulated by a combination of factors, including transcriptional regulation imparted though various tissue-specific and developmental stage-specific signaling pathways, as well as positional cues influenced by morphogen gradients. The developing wing discs of the fruit fly *Drosophila melanogaster* provide an excellent model with which to study mechanisms that control cell fate determination. The adult wing blade pattern includes veins and interveins. There are two types of veins in the wing: longitudinal veins termed L2-L5 and crossveins termed the anterior crossvein (ACV) and the posterior crossvein (PCV). Longitudinal vein primordia appear in the third instar larval stage, and ACV primordia have been reported to appear, although transiently, at this stage as well (Waddington, 1940; Conley et al., 2000). Several signaling pathways regulate wing vein cell fate determination, including EGFR, Hedgehog (HH), DPP and Notch (Sturtevant and Bier, 1995; Sturtevant et al., 1993; Biehs et al., 1998; De Celis, 1997; De Celis et al., 1997; Posakony et al., 1990). EGFR activity drives initial vein-specific gene expression in the larval wing disc, and later maintains vein cell fate specification (through DPP expression) in cells that will become longitudinal veins (Sturtevant et al., 1993; Diaz-Benjumea and Garcia-Bellido, 1990; Guichard et al., 1999; Martin-Blanco et al., 1999; Schnepp et al., 1996; De Celis, 1997). It is known that EGFR signaling is not necessary for the early development of the PCV, but the specific effects of EGFR expression on early development of the ACV and the mechanisms involved are not as well studied.

One way in which EGFR activity controls vein differentiation is through downregulation of the transcription factor Serum response factor (dSRF; also known as Blistered – FlyBase) in longitudinal vein primordia (Roch et al., 1998). dSRF is expressed in third instar larval wing disc cells that are destined to become intervein (Nussbaumer et al., 2000). This expression is then maintained throughout development to eclosure (Montagne et al., 1996). dSRF mutations cause ectopic vein formation, while overexpression of dSRF results in loss of veins, including the ACV (Sturtevant and Bier, 1995; Fristrom et al., 1994; Montagne et al., 1996; Valentine et al., 2014).

Here, we identify a novel role for the Condensin II complex in cell fate determination of third instar larval wing disc cells that will become the ACV. *Drosophila* Condensin II is composed of four subunits, namely SMC2, SMC4 (Gluon – FlyBase), CAP-H2 and CAP-D3, and functions to organize chromatin throughout the cell cycle. Condensin II is essential for the efficient condensation of chromosomes in mitotic prophase. Condensin II also plays important roles in organizing chromosome territories, in preventing homologous chromosome pairing and in organizing topologically associated domains to regulate transcription (Bauer et al., 2012; Hartl et al., 2008; Li et al., 2015; Joyce et al., 2012). The CAP-D3 subunit of Condensin II regulates the transcription of many genes during the larval and adult stages in the fly, including genes involved in cell fate determination (Longworth et al., 2012). Although Condensin II components have been shown to be necessary for the differentiation of mouse ESCs (Dowen et al., 2013), development of T cells (Rawlings et al., 2011) and differentiation of erythroid progenitors (Xu et al., 2006), a role for these subunits in promoting a choice between two cell fates *in vivo* has not been reported.

Here, we show that the cell fate choice to become ACV in the developing wing disc is regulated by CAP-D3 through its ability to maintain EGFR activity in cells immediately anterior to the anterior/posterior (A/P) boundary. This prevents cell death, allowing an EGFR-dependent signal to be transmitted to the neighboring cells in the L3-L4 intervein region, which blocks the upregulation of dSRF and prevents those cells from becoming intervein cells. We show that CAP-D3 represses expression of the KNOT transcription factor in the cells anterior to the A/P boundary, thus alleviating KNOT-mediated repression of EGFR activity. CAP-D3 binds to regions surrounding a *knot* enhancer and helps to maintain repressive histone marks within the region in S2 cells. These data suggest that CAP-D3/Condensin II may regulate enhancer activity to repress *knot* transcription and ultimately influence EGFR-mediated signaling to neighboring cells.

## RESULTS

### Decreased CAP-D3 expression in cells of the developing wing disc results in loss of the ACV and upregulation of dSRF

Gene ontology analysis of published microarray data comparing gene expression levels in whole, wild-type and *Cap-D3* mutant larvae and adults indicated that a significant number of the differentially expressed genes in *Cap-D3* mutants are involved in cell fate determination (Longworth and Dyson, 2010). Currently, null alleles of *Cap-D3* do not exist. Therefore, in an effort to uncover new roles for CAP-D3 during development, double-stranded RNA (dsRNA) against *Cap-D3* was expressed in various tissues and at various stages using different GAL4 drivers (Table 1). The majority of GAL4 drivers used to express *Cap-D3* dsRNA resulted in lethality; drivers that induce expression ubiquitously in all tissues caused early lethality prior to the first instar larval stage, whereas tissue-specific drivers more often caused lethality during the pupal stage.

**Table 1.**
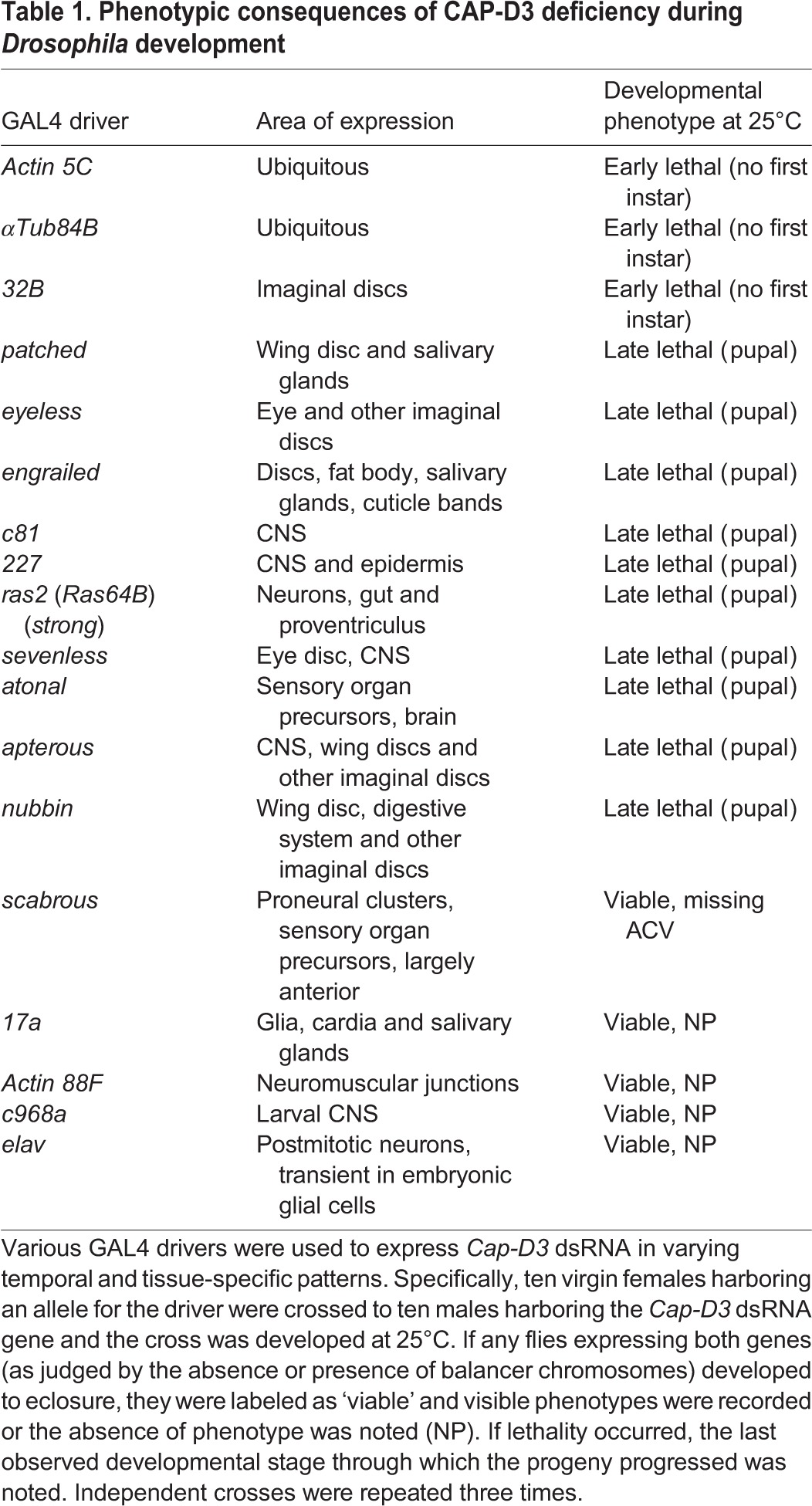
**Phenotypic consequences of CAP-D3 deficiency during *Drosophila* development**

**Fig. 1.**
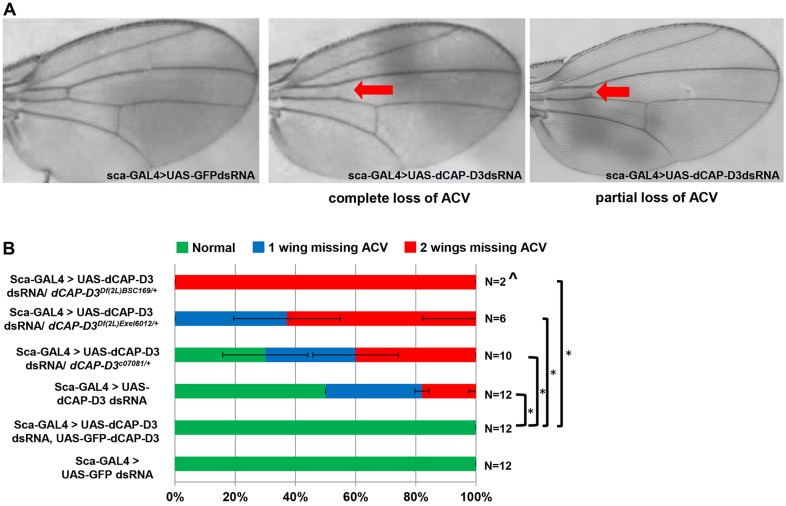
**CAP-D3 deficiency in cells anterior to the anterior/posterior (A/P) border of the developing wing disc results in loss of the anterior crossvein.** (A) Adult *D. melanogaster* wings expressing *Cap-D3* dsRNA under the control of *sca*-GAL4 exhibit complete or partial loss of the anterior crossvein (ACV), as indicated by the red arrow. (B) Frequencies of wings per adult fly that exhibit loss (complete and partial combined) of the ACV. Control flies expressing *GFP* dsRNA are compared with flies expressing various combinations of alleles that are mutant for *Cap-D3*, express *Cap-D3* dsRNA and/or overexpress CAP-D3. Percentages reflect the average of two crosses with *n* total flies in comparison to flies expressing *Cap-D3* dsRNA alone. ^This cross resulted mostly in lethality with analyses performed on *n* escapers. Error bars indicate s.d. *P<0.05 (Fisher's exact test).

Interestingly, *scabrous* (*sca*)-GAL4-driven expression of *Cap-D3* dsRNA in specific regions of the developing wing disc, including sensory organ precursor cells (Singson et al., 1994; Mlodzik et al., 1990), resulted in complete or partial loss of the ACV (Fig. 1A). This phenotype was not completely penetrant and was variable in terms of the number of wings per fly that exhibited loss of the ACV. Approximately 50% of flies expressing *Cap-D3* dsRNA driven by *sca*-GAL4 exhibited loss of the ACV on one or two wings, as compared with control flies expressing *GFP* dsRNA driven by *sca*-GAL4 (Fig. 1B).

Flies homozygous for a hypomorphic mutant allele of *Cap-D3* (*Cap-D3^c07081^*) or transheterozygous for this allele in combination with deletions that encompass the *Cap-D3* locus (*Cap-D3^c07081/Df(2L)Exel6012^; Cap-D3^c07081/Df(2L)BSC169^*) do not exhibit loss of the ACV, whereas single copies of these alleles do increase the frequency of ACV loss when expressed together with *Cap-D3* dsRNA (Fig. 1B). These results show that further decreasing the levels of CAP-D3 expression in the cells of the developing wing disc significantly impacts the development of the ACV, and also impacts the overall fitness of the organism, since crosses including deficiency alleles exhibited more lethality, with escapers developing wings that lacked ACVs (Fig. 1B). Importantly, overexpression of CAP-D3 protein fused to eGFP at its N-terminus [eGFP-CAP-D3 (Longworth et al., 2012)] together with *Cap-D3* dsRNA fully rescued the phenotype in all wings (Fig. 1B). CAP-D3 is a member of the Condensin II complex, which also contains SMC4, SMC2 and CAP-H2. *sca*-GAL4-driven expression of dsRNA targeting the other Condensin II subunits did not cause loss of the ACV (Fig. S1). However, expression of *Cap-D3* dsRNA in flies haploinsufficient for the Condensin II subunit CAP-H2 or the Condensin I/II subunit SMC4 enhanced the frequency of ACV loss (Fig. S2). Expression of *Cap-D3* dsRNA in flies haploinsufficient for Condensin I subunits resulted in a slight enhancement of the phenotype, although not as severe as that seen for CAP-H2 (Fig. S2). These results suggest that the development of the ACV depends most directly on CAP-D3 expression, but is strongly influenced by the Condensin II complex as a whole.

**Fig. 2.**
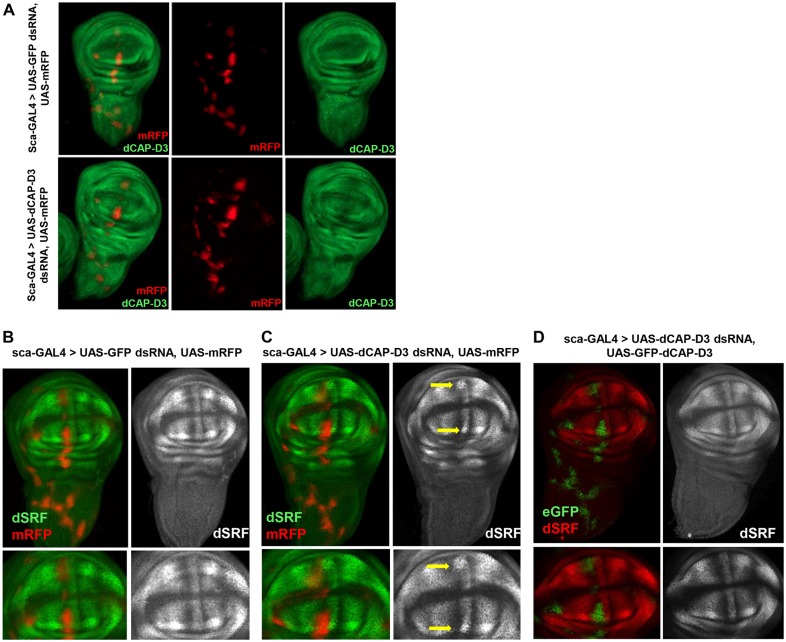
**dSRF expression is increased in cells at the A/P boundary that border CAP-D3-deficient cells in the developing wing disc.** (A) Immunostaining for CAP-D3 (green) in third instar larval wing discs expressing mRFP to mark cells in which *sca*-GAL4 drives expression. Discs expressing *Cap-D3* dsRNA show loss of staining in areas expressing mRFP (bottom row) as compared with control wing discs expressing *GFP* dsRNA (top row). (B) Immunostaining for dSRF (green) in third instar larval wing discs expressing *GFP* dsRNA reveals a normal staining pattern for dSRF. (C) Immunostaining for dSRF (green) in wing discs expressing *Cap-D3* dsRNA shows upregulation of dSRF at the outer edges of both the dorsal and ventral compartments of the pouch, between the L3 and L4 proveins in cells adjacent to those that express mRFP (arrows). (D) Immunostaining for dSRF (red) in wing discs expressing both *Cap-D3* dsRNA and eGFP-tagged CAP-D3 (green) shows rescue of dSRF levels in A/P boundary cells. Discs shown are representative of three experiments on at least five individual larvae per experiment.

To achieve a better understanding of how decreased CAP-D3 expression might lead to loss of the ACV, immunostaining for CAP-D3 protein was performed on third instar larval wing discs expressing *Cap-D3* dsRNA or control *GFP* dsRNA under the control of *sca*-GAL4 (Fig. 2A, Fig. S3A). Myristolated RFP (mRFP) was also expressed in these discs to label the cells in which protein expression was being driven by GAL4. The results showed that CAP-D3 protein expression is effectively knocked down in cells where *sca*-GAL4 drives expression. The efficient knockdown of CAP-D3 by the expressed dsRNA was also demonstrated in third instar larval salivary glands (Fig. S3B).

Interestingly, in the wing disc, cells deficient for CAP-D3 included cells positioned near the A/P boundary, in the region where the ACV is thought to develop (Fig. 2A, Fig. S3A). The A/P boundary is maintained largely through HH-mediated regulation of gene expression (Vervoort, 2000). Two HH targets involved in A/P boundary maintenance are Engrailed (EN) and Cubitus interruptus (CI). EN is expressed on the posterior side of the wing disc and CI is expressed on the anterior side (Vervoort, 2000). Therefore, to determine whether the A/P boundary was disrupted following *Cap-D3* dsRNA expression in cells anterior to the A/P boundary, immunostaining for EN and CI was performed separately (Fig. S4). The results demonstrated that normal EN and CI protein expression patterns were maintained in *Cap-D3* dsRNA-expressing wing discs, as compared with wing discs expressing *GFP* dsRNA, suggesting that no gross changes to the A/P boundary occur as a result of *Cap-D3* dsRNA expression.

Previous work from other labs has shown that dSRF levels in cells at the A/P boundary of the developing wing disc have a significant impact on the development of wing veins, including the ACV (Roch et al., 1998; Montagne et al., 1996; Fristrom et al., 1994). Specifically, overexpression of dSRF leads to loss of the ACV, whereas decreased dSRF expression leads to ectopic wing vein formation between L3 and L4 (Roch et al., 1998). Immunostaining of third instar larval wing discs expressing *Cap-D3* dsRNA revealed a striking upregulation of dSRF in cells at the A/P boundary that were positioned immediately posterior to the mRFP-labeled cells expressing *Cap-D3* dsRNA (Fig. 2B,C, compare yellow arrows). This upregulation was abrogated in wing discs that express eGFP-CAP-D3 in addition to *Cap-D3* dsRNA, suggesting that the increased dSRF levels were caused by CAP-D3 deficiency and were not due to an off-target effect of RNAi (Fig. 2D).

If the loss of the ACV were due to the increased levels of dSRF in cells posterior to *Cap-D3* dsRNA-expressing cells, then knocking down the levels of dSRF in those cells should rescue ACV development. To test this, *sca*-GAL4 was used to drive *Cap-D3* dsRNA in flies heterozygous for a mutant allele of *dSRF* (*bs^3^*) previously shown to exhibit loss-of-function phenotypes similar to other *dSRF* alleles (Donlea et al., 2009) (Fig. S5). The results showed that expression of *bs^3^* did, in fact, suppress the loss of the ACV. These flies, however, also expressed a mutant *plexus* (*px^1^*) allele, and PX has been shown to regulate the expression of many genes involved in wing vein differentiation; *px* mutation increases *rhomboid* (*rho*) expression, which then results in decreased dSRF expression in specific regions of the wing disc (Garcia-Bellido and De Celis, 1992; Matakatsu et al., 1999). Therefore, *Cap-D3* dsRNA was expressed in the background of the mutant *px^1^* allele by itself, but results demonstrated a slight enhancement of phenotype as compared with *Cap-D3* dsRNA expression alone (Fig. S5). Taken together, these data suggest that knockdown of CAP-D3 expression in cells immediately anterior to the A/P boundary causes an upregulation of dSRF that leads to loss of the ACV later in development.

### CAP-D3 deficiency results in decreased EGFR activity

**Fig. 3.**
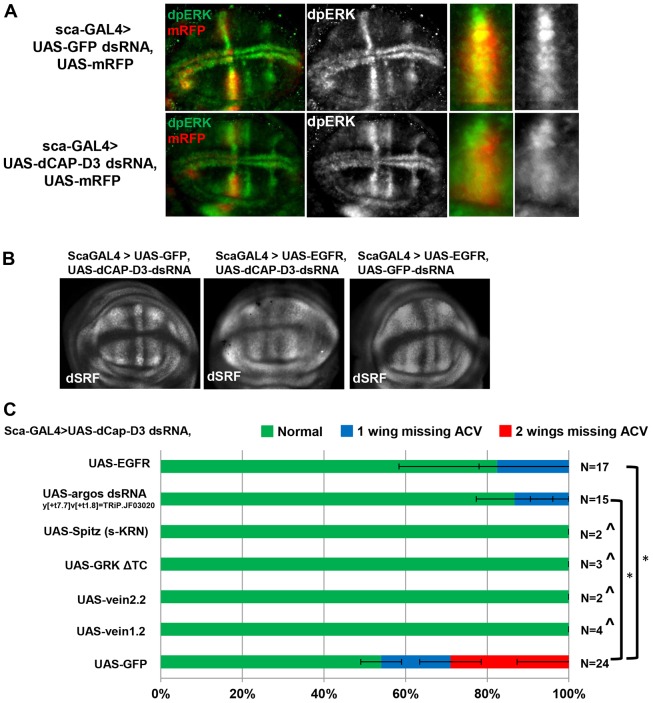
**Increased EGFR activity in CAP-D3-deficient cells decreases dSRF expression at the A/P boundary and rescues ACV development.** (A) Immunostaining for dpERK (green) in wing disc cells expressing *GFP* dsRNA (top row) or *Cap-D3* dsRNA (bottom row) driven by *sca*-GAL4 and marked with mRFP (red) shows lower levels of dpERK in CAP-D3-deficient cells anterior to the A/P boundary. The four panels to the right are magnified images of the staining pattern in the dorsal half of the L3 provein. (B) Immunostaining for dSRF in wing discs expressing *Cap-D3* dsRNA compared with those expressing both *Cap-D3* dsRNA and UAS-EGFR (left versus middle panels) and compared with discs expressing just UAS-EGFR (right panel) under the control of *sca*-GAL4. Discs shown are representative of three experiments on at least five individual larvae per experiment. (C) Frequencies of wings per adult fly that exhibit loss of the ACV in flies expressing *sca*-GAL4-driven *Cap-D3* dsRNA alone or in combination with UAS-EGFR or regulators of EGFR activity. *P<0.05 (Fisher's exact test), in comparison to flies expressing *Cap-D3* dsRNA and UAS-GFP. Error bars indicate s.d. ^This cross resulted mostly in lethality with analyses performed on *n* escapers.

EGF signaling plays a major role in wing vein patterning. EGFR has been shown to repress dSRF levels in the larval wing disc, and loss of EGFR activity results in loss of veins including the ACV (Roch et al., 1998; Price et al., 1989). Since EGFR activation in *Drosophila* results in phosphorylation of MAPK (ERK) at two specific residues (dpERK), and many of the dpERK staining patterns in *Drosophila* imaginal discs (including wing discs) correlate with EGFR activation, immunostaining for dpERK is considered to be an effective readout of EGFR activity in the fly (Gabay et al., 1997). EGFR activity was therefore indirectly measured in *Cap-D3* dsRNA-expressing cells in the wing disc through immunofluorescence analysis of dpERK. The results demonstrated that control *GFP* dsRNA-expressing cells (Fig. 3A, top row, marked with mRFP) exhibit high levels of dpERK staining (green), whereas *Cap-D3* dsRNA-expressing cells exhibit reduced dpERK staining (Fig. 3A, bottom row). Restoration of EGFR activity through overexpression of EGFR in the *Cap-D3* dsRNA-expressing cells in these discs rescued the increased levels of dSRF present in the neighboring A/P boundary cells (Fig. 3B, compare middle and left panels). Overexpression of EGFR in cells expressing control *GFP* dsRNA, however, had no effect on dSRF levels (Fig. 3B, compare right panel with Fig. 2B). In line with these results, overexpression of EGFR in these discs almost completely suppressed the loss-of-ACV phenotype caused by CAP-D3 depletion (Fig. 3C). Additionally, overexpression of secreted forms of various EGFR ligands (Spitz, Gurken, Vein) or expression of dsRNA against an inhibitor of EGFR signaling, Argos, increased lethality in the *Cap-D3* dsRNA-expressing progeny, but rescued ACV development in eclosed flies that escaped lethality (Fig. 3C). Overexpression of a dominant-negative form of EGFR in *Cap-D3* dsRNA-expressing flies resulted in complete lethality, thus making it impossible to evaluate its effects on ACV development (data not shown). These results indicate that decreased levels of CAP-D3 expression in cells adjacent to the A/P boundary result in decreased EGFR activity, which leads to dSRF upregulation and the change to an intervein cell fate decision in the neighboring cells.

### KNOT, a repressor of EGFR activity and activator of dSRF expression, is upregulated in CAP-D3-deficient cells

**Fig. 4.**
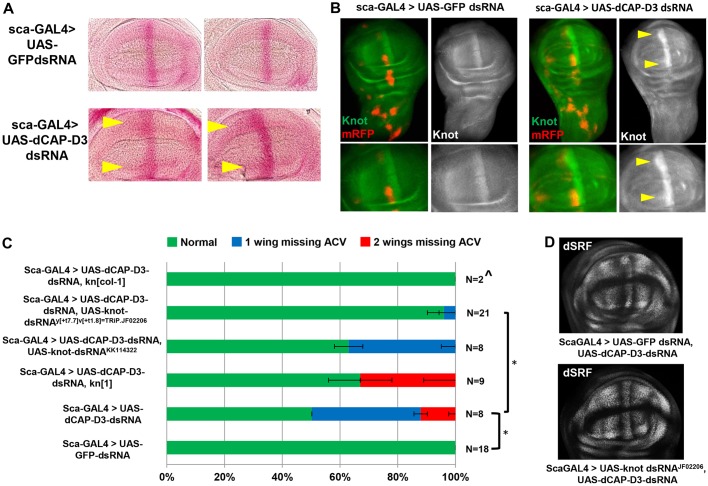
**CAP-D3-mediated repression of *knot* transcription is necessary for maintaining low levels of dSRF at the A/P boundary.** (A) RNA *in situ* hybridization experiments in wing discs expressing *sca*-GAL4-driven *GFP* dsRNA as a control (top row) or *Cap-D3* dsRNA (bottom row) show increased levels of *knot* transcripts in cells anterior to the A/P boundary (arrowheads). (B) Immunostaining for KNOT in wing disc cells expressing *GFP* dsRNA (left panels) or *Cap-D3* dsRNA (right panels) shows increased KNOT protein levels in cells expressing *Cap-D3* dsRNA as marked by mRFP (red) and the arrowheads. (C) Frequencies of wings per adult fly that exhibit loss of the ACV in flies expressing *sca*-GAL4-driven *Cap-D3* dsRNA alone or in combination with *knot* dsRNAs or mutant *knot* alleles. Percentages reflect the average of two crosses with *n* total flies. *P<0.05 (Fisher's exact test), in comparison to flies expressing *Cap-D3* dsRNA alone. Error bars indicate s.d. ^This cross resulted mostly in lethality with analyses performed on *n* escapers. (D) Immunostaining for dSRF in wing discs expressing either *Cap-D3* dsRNA and *GFP* dsRNA (top panel) or *Cap-D3* dsRNA and *knot* dsRNA (bottom panel) demonstrates lower levels of dSRF in the *Cap-D3*/*knot* dsRNA-expressing discs. Discs shown are representative of three experiments on at least five individual larvae per experiment.

KNOT is a helix-loop-helix transcription factor of the COE (Collier/Olf1/EBF) family of transcription factors that has roles in the patterning and development of a number of tissues in the fly. Importantly, KNOT has been shown by several groups to promote intervein cell fate decisions in the wing, largely through its abilities to repress EGFR activity and upregulate dSRF levels (Vervoort et al., 1999; Mohler et al., 2000). Interestingly, *in situ* hybridization experiments in larval wing discs expressing *Cap-D3* dsRNA under the control of *sca*-GAL4, using an antisense probe to detect *knot* transcript levels, revealed that *knot* transcription in third instar larval wing discs is increased in the cells immediately anterior to the A/P boundary, as compared with control wing discs expressing *GFP* dsRNA (Fig. 4A, arrowheads). *In situ* experiments using a control sense probe did not detect any signal (data not shown). Furthermore, KNOT protein is similarly increased in CAP-D3-deficient cells anterior to the A/P boundary, as evidenced by immunofluorescence analyses (Fig. 4B, arrowheads). This increase in KNOT protein in *Cap-D3* dsRNA-expressing cells was quantified by immunostaining for EN to mark the posterior border, followed by measurement of the spread of KNOT protein staining on the dorsal and ventral edges of the wing pouch, in the areas where *sca*-GAL4 drives expression (Fig. S6A, arrows). The distance between the last cell expressing EN in the posterior compartment and the last cell expressing KNOT in the anterior compartment was measured in the regions at the distalmost portions of the pouch, and then divided by the same measurement taken in the middle of the pouch at the regions on each side of the dorsal/ventral boundary, to control for changes in disc shape. Results showed a significant increase in the spread of KNOT staining anterior to the A/P boundary on the dorsal half of wing discs expressing *Cap-D3* dsRNA (Fig. S6B). There was also an average increase in KNOT staining anterior to the A/P boundary on the ventral half of these discs, but it was more variable between discs, and therefore not as statistically significant (Fig. S6C).

To test whether this upregulation of KNOT in CAP-D3-deficient cells anterior to the A/P boundary might be responsible for increased dSRF levels in the neighboring cells and the concomitant loss of the ACV, *sca*-GAL4 was used to drive *Cap-D3* dsRNA in combination with *knot* dsRNA or in flies that were heterozygous for a hypomorphic allele of *knot*, *kn[1]*, or the amorphic allele *kn[col-1]* (Fig. 4C). Expression of *Cap-D3* dsRNA in cells that also expressed the *kn[col-1]* allele resulted in complete rescue of ACV development, although the number of progeny that eclosed was drastically reduced. Expression of *knot* dsRNA or the *kn[1]* allele also suppressed the loss-of-ACV phenotype, although not to the same extent as the *kn[col-1]* allele. Co-expression of *Cap-D3* dsRNA and *knot* dsRNA, which suppressed the loss-of-ACV phenotype to the greatest extent (*knot dsRNA^y[+t7.7]v[+t1.8]=TRiP.JF02206^*), also depressed dSRF in cells in the A/P boundary to levels seen in control wing discs expressing *GFP* dsRNA (Fig. 4D).

### CAP-D3 binds to DNA surrounding the KN01 enhancer in the *knot* locus

**Fig. 5.**
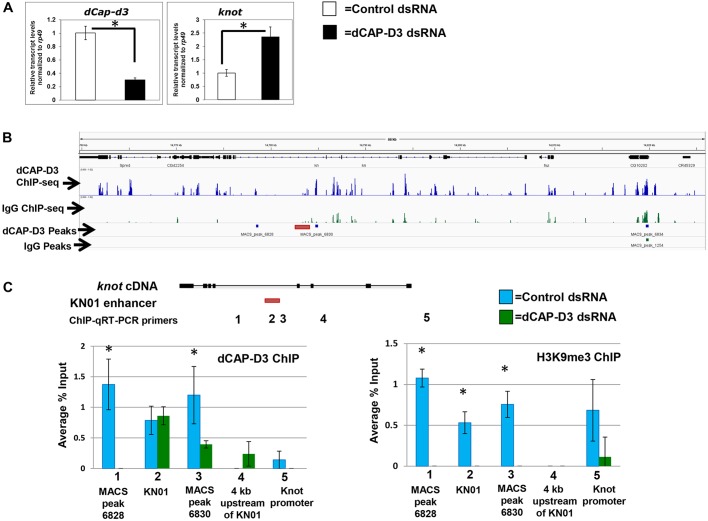
**CAP-D3 binds the region encompassing the *knot* enhancer KN01 and represses *knot* transcription in S2 cells.** (A) qRT-PCR analysis of *Cap-D3* and *knot* transcript levels in S2 cells shows that *Cap-D3* dsRNA treatment decreases *Cap-D3* transcripts and increases *knot* transcripts in comparison to treatment with control (T7) dsRNA. Data are the average of three experiments. (B) Depiction of ChIP-seq results at the *knot* locus, showing significant CAP-D3 peaks (blue) not found in the set of IgG ChIP-seq peaks (green). All peaks were first normalized to input and significant peaks were identified by MACS peak finder (bottom two rows, where boxes indicate a significant peak). (C) ChIP for CAP-D3 (left) or H3K9me3 (right) was performed in S2 cells treated with control or *Cap-D3* dsRNA and analyzed by qRT-PCR. The location of the KN01 enhancer is depicted by a red box. Sequences targeted by primer sets for the ChIP-qRT-PCR experiments are indicated (1-5). *P<0.05, Student's unpaired *t*-test; error bars indicate s.d.

The expression of *knot* in the developing wing disc is influenced by many signaling pathways, but transcription in the wing pouch of the third instar larval wing disc is directly controlled by two different enhancers (Hersh and Carroll, 2005; McKay and Lieb, 2013). The more recently identified KN01 enhancer seems to be more active in the pouch, and is located in the fourth intron of the gene (McKay and Lieb, 2013). Since our data suggested that CAP-D3 represses *knot* transcription, we tested whether it does so directly in S2 cells. qRT-PCR analysis demonstrated that decreased *Cap-D3* levels in S2 cells expressing *Cap-D3* dsRNA did indeed result in upregulation of *knot* transcripts, similar to observations in the wing disc (Fig. 5A). These analyses also revealed that *dSRF* levels remain unchanged in *Cap-D3* dsRNA-expressing S2 cells (Fig. S7). This is not surprising, however, given that EGFR protein is not expressed in S2 cells (Schweitzer et al., 1995) and therefore does not act to repress *dSRF* expression, thus rendering *knot* upregulation irrelevant.

Chromatin immunoprecipitation for CAP-D3 combined with high-throughput sequencing (ChIP-seq) was next performed in S2 cells and compared with ChIP-seq for IgG as a control, following normalization of both data sets to the input signal. In total, 9059 binding sites (*P*=1×10^−10^) were identified for CAP-D3 (see GEO accession GSE74413). Previously, it was shown that CAP-D3 orthologs in other organisms bind primarily to gene promoters (Kranz et al., 2013). However, the majority of CAP-D3 binding sites in S2 cells were not within gene promoters, with more than 50% of the binding sites positioned within exons and introns (Fig. S8).

Upon further inspection of the CAP-D3 ChIP-seq data, it was determined that two significant CAP-D3 binding sites flank the KN01 enhancer sequence (Fig. 5B). To confirm that these sites are bound by CAP-D3 in S2 cells, ChIP with CAP-D3 antibody and qRT-PCR analyses using primers that spanned the *knot* locus (including the potential CAP-D3 binding sites) were performed in control or *Cap-D3* dsRNA-treated cells (Fig. 5C, left panel). Results confirmed that CAP-D3 binding to both of the identified sites (MACS peaks 6828 and 6830) was significantly depleted in *Cap-D3* dsRNA-treated cells (Fig. 5C). Interestingly, examination of the top 1000 peaks found outside promoters for common CAP-D3 binding motifs (using the MEME-ChIP web server) revealed that nine motifs were significantly enriched at CAP-D3 binding sites and that one motif, AA(G/A)TGG, is present in the confirmed CAP-D3 binding site corresponding to MACS peak 6830 (sequence not shown).

Given that CAP-D3 has been shown to flank and play a role in the maintenance of repressive chromatin marks of other genes that it transcriptionally represses (Schuster et al., 2013), ChIP analyses for the histone H3 trimethylated lysine 9 (H3K9me3) repressive mark were performed in control and *Cap-D3* dsRNA-treated cells. Results showed that H3K9me3 is present in the region bound by CAP-D3, which surrounds and encompasses the KN01 enhancer (Fig. 5C, right panel). Furthermore, H3K9me3 marks are significantly depleted at this region in *Cap-D3* dsRNA-treated cells, suggesting that CAP-D3 might act to maintain this repressive chromatin mark within the region, potentially promoting a more closed chromatin configuration that might prevent interaction of the KN01 enhancer with the promoter. Finally, ChIP analyses for histone H3 acetylated at lysine 27 (H3K27ac, a mark found at active enhancers) demonstrated no significant changes in distribution within the region in *Cap-D3* dsRNA-treated cells, suggesting that CAP-D3 is not necessary for maintaining H3K27ac at KN01 (Fig. S9). Together, these data suggest that CAP-D3 may directly repress transcription of the *knot* gene by binding to sites that flank the KN01 enhancer and maintaining repressive chromatin marks within the region.

### CAP-D3 represses *knot* and maintains EGFR activity to prevent cell death and promote non-cell-autonomous repression of dSRF

**Fig. 6.**
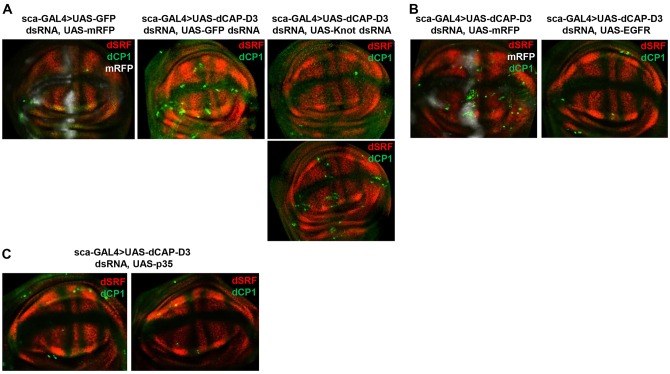
**CAP-D3 deficiency in cells anterior to the A/P boundary results in cell death.** (A) Immunostaining for dSRF (red) and DCP-1 (dCP1, green) show variably elevated levels of DCP-1 in cells anterior to the A/P boundary in discs expressing *Cap-D3* dsRNA as compared with discs expressing *GFP* dsRNA (compare middle and leftmost panels). DCP-1 staining in discs expressing both *Cap-D3* dsRNA and *knot* dsRNA more closely resembles that of control discs (compare rightmost and leftmost panels). (B) Immunostaining for dSRF (white) and DCP-1 (green) in wing discs expressing EGFR and *Cap-D3* dsRNA (right panel) show decreased levels of DCP-1 in comparison to discs expressing *Cap-D3* dsRNA alone (left panel). (C) Immunostaining for dSRF (red) and DCP-1 (green) in wing discs expressing p35 (the baculovirus inhibitor of caspase) and *Cap-D3* dsRNA show decreased levels of DCP-1 in comparison to discs expressing *Cap-D3* dsRNA alone (compare with B, left panels). Discs shown are representative of three experiments on at least five individual larvae per experiment.

EGFR activity is important for preventing cell death in a number of organisms and tissues (Doroquez and Rebay, 2006; Bergmann et al., 1998). Interestingly, *sca*-GAL4-driven expression of *Cap-D3* dsRNA resulted in a significant decrease in the distance between the outer edges of L3 and L4 in the adult wing compared with the *GFP* dsRNA control [27.41±3.71 (s.d.) versus 28.82±2.94; *n*=44 each; *P*<0.05, Student's unpaired *t*-test]. This indicated that cell death might occur earlier in development in some of the cells in this area. To further examine whether the role of CAP-D3 in promoting EGFR activity is also necessary to prevent cell death, *Cap-D3* dsRNA was expressed using the *sca*-GAL4 driver and wing discs were immunostained for dSRF and DCP-1 (Death caspase-1), a *Drosophila* member of the caspase family of ICE/CED-3 proteases. Results demonstrated that *Cap-D3* dsRNA expression does increase the number of DCP-1-positive cells that lie immediately anterior to the cell exhibiting increased levels of dSRF in the A/P boundary (Fig. 6A). Furthermore, increases in DCP-1 are abrogated when *knot* dsRNA or UAS-EGFR is expressed in combination with *Cap-D3* dsRNA (Fig. 6A,B), although the rescue of cell death is somewhat variable, and discs that exhibit some residual DCP-1 in the pouch also exhibit increased dSRF in the neighboring areas (Fig. 6A, bottom right panel, dorsal side of wing disc). Cell death was largely unaffected in the notum of the majority of these discs (Fig. S10). These findings suggest that CAP-D3 repression of *knot* in cells anterior to the A/P boundary maintains EGFR activity to promote cell survival.

To determine whether rescue of cell death in CAP-D3-deficient cells was sufficient to prevent the upregulation of dSRF, the baculovirus inhibitor of caspase, p35, was expressed in combination with *Cap-D3* dsRNA and discs were again stained for DCP-1 and dSRF. The increased levels of DCP-1 were abrogated, as expected (Fig. 6C). The results however showed that increased levels of dSRF remain, as compared with *sca*-GAL4-driven *Cap-D3* dsRNA expression alone (compare Fig. 6C with 6A). Since CAP-D3 levels and EGFR activity remain low in cells expressing p35 that were rescued from cell death, these data indicate that CAP-D3 and EGFR activity is also necessary for the non-cell-autonomous signaling that occurs between the cells anterior to and the cells within the A/P boundary that is responsible for blocking the expression of dSRF.

## DISCUSSION

### CAP-D3/Condensin II-mediated regulation of cell fate in the wing disc

**Fig. 7.**
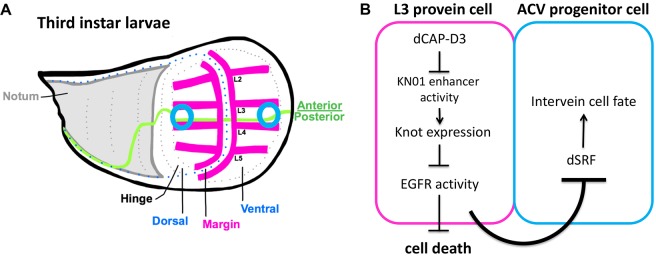
**Model of the mechanism by which CAP-D3 regulates cell fate determination in cells destined to become ACV progenitor cells.** (A) Diagram of the third instar larval wing disc. Blue circles represent the regions from which ACV progenitor cells develop and pink lines represent longitudinal vein precursor cells. (B) In the model, CAP-D3 represses *knot* transcription, potentially by binding to the KN01 enhancer and preventing its ability to activate *knot* transcription in the L3 provein cells just anterior to the ACV progenitor cells present in the A/P boundary. This repression of *knot* expression maintains EGFR activity in these cells, preventing cell death and facilitating an EGFR-dependent signal that acts to dampen dSRF expression in neighboring ACV progenitor cells. This keeps dSRF levels balanced with other regulators of cell fate determination, allowing the cells to become ACV.

The *Drosophila* wing represents an excellent model with which to study signaling pathways and transcriptional programs that influence cell fate decision and, more specifically, the differentiation of cells into vein or intervein. The ACV and PCV begin to develop structurally during the pupal stage, but ACV development is largely influenced by the expression of dSRF and its regulators during the third instar larval stage (Montagne et al., 1996; Roch et al., 1998). The data presented here show that CAP-D3 expression in cells anterior to the A/P boundary (pink anterior cells on the ventral and dorsal side, Fig. 7A) prevents the upregulation of dSRF in a non-cell-autonomous manner in the two neighboring regions positioned between L3 and L4 at the dorsalmost and ventralmost areas of the pouch (Fig. 7A, blue circles). The data suggest that CAP-D3 binds sites surrounding the KN01 enhancer and represses the transcription and expression of *knot*. This maintains EGFR activity in the cells anterior to the A/P boundary, thus promoting cell survival and facilitating an unidentified EGFR-dependent signal that travels to the neighboring A/P boundary cell to dampen dSRF expression and block the cell fate decision to become an intervein cell (Fig. 7B). This novel mechanism by which CAP-D3 promotes development of the ACV is the first report of the involvement of a Condensin II subunit in regulating cell fate decisions *in vivo*. The data presented here show that other Condensin II subunits may play a role in this regulation, as their downregulation in combination with CAP-D3 enhances the loss-of-ACV phenotype (Fig. S2). However, since individual knockdown of the other subunits does not cause the phenotype it is possible that either CAP-D3 levels are rate limiting for the proposed pathways or that the dsRNAs used to knock down the other subunits are inefficient. The latter is currently hard to test, given the lack of available antibodies that can be used for this purpose.

### Maintenance of EGFR activity by CAP-D3

EGFR activity is important for the specification of many different cell types throughout fly development. To our knowledge, this is the first report of a Condensin complex functioning to maintain EGFR signaling during development. In an effort to understand whether the ability of CAP-D3 to regulate EGFR activity exists in other cell types, CAP-D3 levels were altered in ovarian follicle cells. It is well known that EGFR activation is required for the establishment of the dorsal/ventral axis of the egg and the embryo (Queenan et al., 1997; Price et al., 1989). Expression of an activated form of EGFR (lambda top) in the follicle cells of the ovary induces a dorsal cell fate in the developing egg, whereas expression of a dominant-negative form of EGFR induces a ventral cell fate. Therefore, to determine whether altering the levels of CAP-D3 would have similar effects, we expressed UAS-GFP-CAP-D3 or *Cap-D3* dsRNA in the follicle cells using the T155-GAL4 driver (Hrdlicka et al., 2002) and examined eggs laid by these females. Although we did observe the expected phenotypes caused by overexpression of constitutively active or dominant-negative EGFR, we did not see similar morphological changes due to overexpression or underexpression of CAP-D3 (Fig. S11). These data suggest that the ability of CAP-D3 to promote EGFR activity is not functional in all cells in the fly and might be very tissue specific. It is also possible that CAP-D3 only promotes EGFR activity in tissues where KNOT is expressed and involved in the repression of EGFR.

A major question that develops from these studies is the identity of the signal that is elicited by cells just anterior to the A/P boundary and which acts on the cells in the A/P boundary to potentiate cell fate decisions. One possibility is that the EGFR ligand Spitz is responsible for the signaling between the cells, and that in CAP-D3-deficient cells there might be a defect in Spitz localization. It was recently shown that the localization of transmembrane Spitz to the apical region of imaginal disc cells renders it incapable of activating EGFR in neighboring cells (Steinhauer et al., 2013). Both overexpression of secreted Spitz and knockdown of Argos, which associates predominantly with Spitz to prevent EGFR signaling (Vinos and Freeman, 2000), rescue the loss-of-ACV phenotype in CAP-D3-deficient cells (Fig. 3C).

Interestingly, cell adhesion can also play a major role in potentiating the EGFR signal from one cell to the next. RAP1, a small GTPase that regulates DE-cadherin (Shotgun)-mediated cell adhesion, indirectly maintains EGFR signaling in the wing disc through these contacts, and *Rap1* mutant tissue exhibits planar cell polarity defects and loss of wing vein differentiation (O'Keefe et al., 2009). Although we have not formally tested the idea, it is possible that CAP-D3 deficiency anterior to the A/P boundary results in loss of cell-cell adhesion, thus preventing transduction of the EGFR signal into the A/P boundary cells.

### Potential mechanisms by which CAP-D3 may regulate the KN01 enhancer

Maintenance of the EGFR signal in cells anterior to the A/P boundary seems to rely on the ability of CAP-D3 to repress *knot* (Fig. 4). Our data suggest that CAP-D3 may repress *knot* through binding to regions surrounding the KN01 enhancer and maintaining H3K9me3 (Fig. 5). H3K9me3 is found at repressed chromatin, and more recently was shown to be present at ‘poised’ enhancers in mouse ESCs (Zentner et al., 2011); active enhancers in these cells were found to lose the H3K9me3 mark. Given that Condensins are implicated in the regulation of DNA looping *in vitro* and in *Drosophila* tissue culture cells (Bazett-Jones et al., 2002; Kimura et al., 1999; Li et al., 2015), it is possible that CAP-D3 regulates looping at the KN01 enhancer to maintain a repressed, yet poised, chromatin state.

An analysis of chromatin marks present in the region, using modENCODE (www.modencode.org; Kharchenko et al., 2011), suggests that a large Polycomb-repressed domain exists over the entire *knot* locus but, interestingly, that open chromatin appears toward the 3′ end. This suggests that a border between transcriptionally active and repressed chromatin might exist very close to the KN01 enhancer. As Condensin II has been shown to bind to these types of borders and to help to organize topologically associated domains (TADs), it is possible that CAP-D3 binding to KN01 functions in this capacity (Van Bortle et al., 2014; Li et al., 2015). Furthermore, disruption of TADs can alter long-range interactions between enhancers and promoters or other gene regulatory elements, resulting in gene misexpression and serious developmental abnormalities (Lupianez et al., 2015). Future chromosome conformation capture (3C) assays will be necessary to test these ideas.

### Broader implications of CAP-D3-mediated *knot* repression

KNOT expression is important for *Drosophila* development in several contexts. It regulates head development (Crozatier et al., 1996, 1999), is important for embryonic muscle cell fate specification (Crozatier and Vincent, 1999; Dubois et al., 2007) and regulates hematopoietic progenitor cell fate (Pennetier et al., 2012; Benmimoun et al., 2015). KNOT is expressed in postmitotic neurons and controls class IV-specific dendritic arbor morphology in the *Drosophila* peripheral nervous system (Crozatier and Vincent, 2008; Hattori et al., 2013; Jinushi-Nakao et al., 2007). KNOT is also necessary (in combination with other transcription factors) for cell fate specification of specific neurons in the ventral nerve cord and is part of a feed-forward cascade that activates specific terminal differentiation genes in specific neurons (Baumgardt et al., 2007). Overexpression of KNOT causes misexpression of major neuropeptides and drives the cell toward one fate over another (Baumgardt et al., 2009). The question then arises as to whether CAP-D3 might contribute to cell fate specification programs in specific neurons and/or other tissues through its ability to repress *knot* transcription. The fact that *Cap-D3* dsRNA expression mediated by some GAL4 drivers that drive expression in the CNS results in pupal lethality (Table 1), suggests that CAP-D3 might play an important role in the development of specific cells in the CNS and is an idea that needs to be more thoroughly explored.

Recently, it was shown that a high sugar diet promotes O-linked N-acetylglucosamine (O-GlcNAc) transferase (OGT)-mediated O-GlcNAc modification of Polyhomeotic, leading to Polycomb-mediated upregulation of *knot* in *Drosophila* pericardial nephrocytes, which are analogous to the mammalian podocyte (Na et al., 2015). Podocytes encircle the glomerular capillaries, and reorganization of their actin cytoskeleton in diabetic patients can lead to albuminuria (Reidy et al., 2014). Upregulation of *knot* in response to the high sugar diet caused downregulation of the Nephrin-like protein SNS in *Drosophila* nephrocytes and resulted in phenotypes similar to those seen in patients with diabetic nephropathy (Na et al., 2015). The mammalian orthologs of KNOT are the Early B-cell factor (EBF) proteins EBF1-4 and the Na et al. (2015) study also showed that nuclear EBF2 levels were increased in both human patients and mouse models of diabetic kidney disease. If the novel pathway that we describe here is conserved in human cells, with CAP-D3 (NCAPD3)/Condensin II mediating repression of EBF transcription factors, then CAP-D3 may represent a novel therapeutic target, the facilitated upregulation of which might help to remedy functional defects in podocytes exhibited by diabetic patients.

## MATERIALS AND METHODS

### *Drosophila* stocks and cell lines

The following stocks were obtained from the Vienna Drosophila RNAi Library: *UAS-Cap-D3-dsRNA^GD913^*, *UAS-Cap-H2-dsRNA^KK103162^*, *UAS-SMC2-dsRNA^GD4489^*, *UAS-SMC2-dsRNA^KK100466^*, *UAS-SMC4-dsRNA^GD4454^* and *UAS-knot-dsRNA^KK114322^*. The following stocks were obtained from the Bloomington Drosophila Stock Center at Indiana University: *px[1] bs[3],UAS-Egfr* (*w[+mc]=UAS-Egfr.B*), *Egfr* dominant-negative (*w[+mc]=UAS.Egfr.DN.B*), *argos* dsRNA stock (*y[+t7.7]v[+t1.8]=TRiP.JF03020*), *knot* dsRNA (*y[+t7.7]v[+t1.8]=TRiP.JF02206*), *UAS-knot* (*w[+mc]=UAS-kn.M*) and all GAL4 driver stocks. The *kn[1]* stock was obtained from the Kyoto Drosophila Genetic Resource Center. Other stocks used were: *UAS-sKer* (Urban et al., 2002), *UAS-GrkΔTC* (Queenan et al., 1999), *UAS-vn1.1* and *UAS-vn1.2* (Schnepp et al., 1998) and *kn[col-1]* (Crozatier and Vincent, 1999). All flies were maintained on standard dextrose medium. All crosses were performed at 25°C. S2 cells were obtained from the Drosophila Genomics Resource Center at Indiana University (where they are termed S2-DRSC).

### Immunofluorescence

Third instar larval wing discs were dissected in PBS on ice for 15 min then fixed with 4% paraformaldehyde and 0.5% Triton X-100 for 25 min with rocking at room temperature. Discs were washed twice in PBT (0.1% Triton X-100 in PBS) plus 1% BSA (PBT-BSA) before blocking for 2 h at room temperature in PBT-BSA buffer. Primary antibodies were diluted in PBT-BSA and discs were incubated overnight at 4°C. Discs were washed five times in PBT for 5 min each at room temperature with rocking. All secondary antibodies were diluted 1:500 in PBT-BSA and incubated with discs for 2 h at room temperature with rocking. Discs were washed five times in PBT before two 5 min washes in 0.3% Tween 20 in PBS. Discs were mounted in VectaShield with DAPI (Vector Labs). Imaging was performed on a Zeiss AxioImager Z1 motorized epifluorescence microscope using an MRm CCD camera with a 20× objective lens. Antibodies are listed in the supplementary Materials and Methods.

### RNA *in situ* hybridization

Probes were designed as described by Zimmerman et al. (2013) with slight modifications using *knot* cDNA clone RE03728 (Drosophila Genomics Resource Center at Indiana University). RNA *in situ* hybridization using sense (not shown) and antisense probes was performed as described by Su et al. (2011) and developed using the Vector Red Alkaline Phosphatase Substrate Kit (Vector Laboratories). Images were taken on a Leica SCN400 slide scanner.

### dsRNA treatment and qRT-PCR analysis

dsRNA treatments and qRT-PCR analyses were performed as described (Schuster et al., 2013). Primers are listed in the supplementary Materials and Methods.

### ChIP and ChIP-seq analyses

ChIP experiments were performed as described (Schuster et al., 2013). DNA used in both ChIP and ChIP-seq experiments was sheared with 500 units of micrococcal nuclease per tube containing nuclei from 5×10^7^ cells. 10 µg CAP-D3 antibody YZ384 (Longworth et al., 2012) or 10 µg IgG antibody was used for immunoprecipitations. Three separate ChIP experiments were performed for CAP-D3 and IgG for both ChIP-qRT-PCR and ChIP-seq. DNA samples used in ChIP-seq were quantitated by qubit (Life Technologies) and ChIP-seq libraries were prepared at the University of Chicago Genomics Core (input 5-10 ng DNA) using the Illumina TruSeq ChIP Sample Prep Kit (IP-202-1012). Library quantity and quality was assessed by a Bioanalyzer (Agilent). The pool of libraries was sequenced using the Illumina HiSeq 2500. Data analysis is described in the supplementary Materials and Methods. High-throughput sequencing data described in this manuscript have been deposited at GEO under accession GSE74413.

### Measurement of width of the L3-L4 intervein region

Adult wings were dissected, mounted in Euparal Mounting Medium (Bioquip Products, 6372A) and then imaged on a Leica DFC3000 G stereo fluorescence microscope. The width of the L3-L4 intervein region, from outer edge to outer edge, was measured using arbitrary units in Adobe Photoshop (1 unit=0.085 mm).
